# Landolt C acuity follows Bloch's Law for short-duration flashes

**DOI:** 10.3389/fnins.2026.1954783

**Published:** 2026-09-25

**Authors:** Constantin C. Ketz, Michael Bach, Sven P. Heinrich

**Affiliations:** Eye Center, Medical Center – University of Freiburg, Faculty of Medicine, University of Freiburg, Freiburg, Germany

**Keywords:** Bloch's Law, flash strength, presentation duration, shape recognition, temporal summation, time-intensity reciprocity, visual acuity

## Abstract

**Purpose:**

Bloch's Law states that up to a critical duration, typically between 30 and 100 ms, the detection of a flash depends on its total strength (the product of luminance and duration). This study investigated whether this principle of temporal summation extends to shape recognition, assessed via optotype-based visual acuity.

**Methods:**

Monocular Landolt-C acuity was measured in 19 healthy participants across 25 stimulus conditions. These conditions combined five photopic luminance levels (3.3–53 cd/m^2^) with five presentation durations ranging from 0.125 to 512 ms. The combinations were grouped into five flash strength levels, spanning a 256-fold range from 6.7 to 1,708.2 cd · s/m^2^ × 10^−3^.

**Results:**

Across Flash Strength Levels 1 to 4, mean visual acuity remained nearly constant within each level, with deviations below 0.08 logMAR (less than one line on a typical acuity chart). In contrast, at Level 5 (32–512 ms), visual acuity improved significantly by two lines, with the strongest increase occurring around 100 ms.

**Conclusion:**

The results demonstrate that a “Bloch-like” law also governs shape recognition. Across four octaves in luminance and three orders of magnitude in duration, reductions in luminance were effectively compensated by longer presentation times and vice versa.

## 1 Introduction

Visual acuity measurement is a standard ophthalmological procedure used to assess visual function and monitor disease progression. It is typically performed without restricting the inspection time of the optotype. However, limiting inspection time is often appropriate, as it prevents the use of cognitive strategies and ensures that the measurement primarily reflects visual rather than cognitive processes. In addition, extended viewing is unrepresentative of dynamic real-world demands, such as during driving. Very short optotype inspection durations, in the order of 100 ms, also occur when fast stimulus sequences are used for objective, event-related-potential-based testing of acuity (Gopiswaminathan et al., 2024).

When the presentation duration is reduced below a critical value, the perceived brightness is also altered despite equal luminance, introducing an additional factor that influences the measurement outcome. This is described by Bloch's Law, which relates duration (*t*) and luminance (*L*) required for the detection of a light stimulus (Bloch, 1885; Gorea, 2015). It states that the product of luminance and duration at the detection threshold θ is a constant:


$$\begin{array}{l} {\left(L\cdot t\right)}_{\theta }=\text{const. if }t<t_{\text{c}}. \end{array}$$

This means that a shorter stimulus with higher luminance can be detected with the same probability as a longer stimulus with lower luminance. This only applies to stimuli shorter than the critical duration (*t*_c_) which is approximately 30 ms for standard achromatic perimetric stimuli (Mulholland et al., 2015). Most recently, Bloch's Law was re-evaluated by measuring contrast thresholds for simple geometric shapes across microsecond to millisecond exposures using an LCD/LED tachistoscope (Amerio and Cleeremans, 2025). Across five tasks, the critical duration was in the range of about 15–36 ms. It is important to understand that the critical duration is not constant: it can be influenced by factors such as background luminance (Barlow, 1958), since higher adaptation levels lead to shorter summation periods, whereas lower (scotopic) levels are associated with longer durations. These general stimulus characteristics, together with task-relevant factors such as optotype size and the resulting visibility, collectively influence the critical duration (Kahneman, 1964).

In keeping with Robson et al. (2022), we use the term flash strength (FS) to denote the product of luminance *L* (in cd/m^2^) and duration *t* (in seconds):


$$\begin{array}{l} \text{FS}=L\cdot t\;\left[\text{cd}\cdot \text{s}/{\text{m}}^{2}\right], \end{array}$$

Subsequently, “visual acuity” refers specifically to the measured test outcome under given conditions, rather than the abstract construct of acuity as an intrinsic individual trait or to the broader concept of a person's visual ability.

Physiologically, temporal summation serves to optimize the signal-to-noise ratio through neural integration while also reflecting the inherent dynamics of the underlying physiological processes. This process begins at the level of the photoreceptors, where the temporal response is constrained by the biochemical kinetics of phototransduction (Yau, 1994). Additional summation occurs at the post-receptoral level: retinal ganglion cells integrate signals from upstream bipolar cells over a specific temporal window (Meister and Berry, 1999), thereby enhancing the detection probability for weak stimuli. While these mechanisms are well-characterized for simple light detection, shape recognition (such as optotype identification) likely involves higher cortical processing stages. There, dynamics are more complex and further extend the integration duration. The temporal characteristics of higher-level visual tasks, therefore, cannot be straightforwardly predicted from data obtained solely from simple light detection tasks (Wang, 2012).

Bloch's Law has served as a fundamental principle for describing the temporal summation of luminance flashes for more than a century. However, it has remained uncertain to what extent it applies to shape recognition, which likely depends on more complex neural mechanisms with diverse temporal characteristics. Given that standard Landolt-C optotypes target recognition acuity rather than resolution acuity (Heinrich and Bach, 2013), they provide a useful tool for investigating the temporal dynamics of the multi-stage process underlying shape recognition (Loffler, 2008).

Kahneman (1964) used a Landolt-C acuity task to determine the critical duration up to which temporal summation occurs. He found that, in the range from 50 to 1,000 ms, it varied non-monotonically with the flash strength of the stimulus; furthermore, the acuity level determined the boundaries for temporal summation.

For extremely short flashes in the microsecond range, Greene and Ogden (2013) showed that luminance-duration reciprocity does not apply, indicating that visual perception is not determined solely by photon count. Greene (2013) further demonstrated a “violation of Bloch's Law” in this regime for both shape recognition and flash detection. Overall, these studies suggest a lack of agreement regarding the validity range of Bloch's Law. Our goal was to use very short stimulus durations combined with low luminances to gain new insights into temporal summation for shape recognition around the size threshold.

## 2 Materials and methods

### 2.1 Participants

Twenty participants were recruited. Of these, 19 participants (nine female and 10 male) met all inclusion criteria of no self-reported ophthalmological, neurological, or psychiatric disorders. As an additional inclusion criterion, the mean visual acuity across all four reference measurements was required to be better than 0.1 logMAR. The age range was 20–30 years (mean 23.2 years, SD = 2.76 years). All measurements were performed monocularly to avoid binocular summation and other interocular interactions, with the tested eye being freely chosen by the participants. Ocular dominance was not assessed. The contralateral eye was patched. Four participants wore their habitual correction. All participants provided written informed consent. The study was part of a series of studies that was approved by the local institutional review board.

### 2.2 Set-up

#### 2.2.1 Hardware

The stimuli were displayed on a 21.5-inch, 1920 × 1080 px transparent LCD monitor (*Transparent Display Integration Kit 21.5 LCD, TRN Crystal Display Systems Ltd., Rochester, United Kingdom*), which lacked the backlight normal LCD monitors have. Instead, the monitor was illuminated from behind by an LED panel (*I-Lumen 200* × *100 mm, Amazon ASIN B07GB6TZ4W*) covering an area of 8.2 × 8.2 cm (1.18° visual angle) (Figure 1) and driven by a Raspberry Pi computer. This allowed for very short stimulus presentation durations, much shorter than with a conventional monitor, down to 0.125 ms in the present study. At the corners of this illuminated square were four red LEDs, which guided fixation and remained lit at all times to facilitate accommodation to the correct distance. The front of the monitor was covered by black opaque cardboard with a 14.5 × 14.5 cm (2.08° visual angle) viewing aperture, in which a 0.9-log ND filter (*ND-Filter 8*×*, Haida 150 Pro II ND 0.9, Ningbo Haida Photo Supplies Co., Ltd, China*) was fitted. The participants sat 4 m away from the monitor, which was mounted at a height of 1.10 m, placing the center of the presentation field approximately at eye level. Responses were entered using a numeric keypad with keys assigned topographically to represent the eight possible gap positions of the Landolt C. Additional keys allowed for pausing and continuing the experiment. Starting one minute before the test runs, the ceiling lighting was dimmed to a room brightness of 3 lux. The low illumination ensured large pupils without mydriasis.

**Figure 1:**
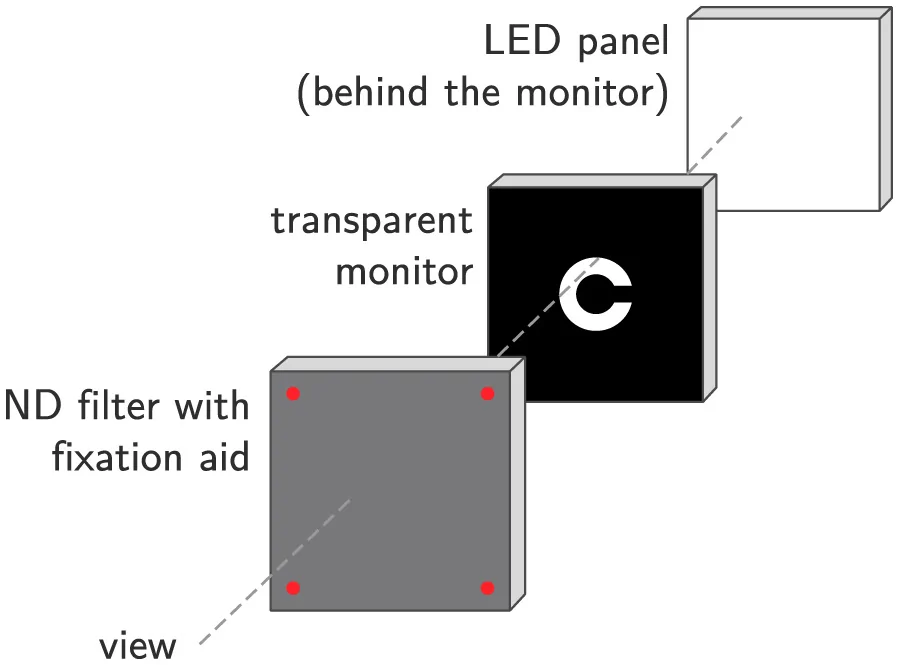
Experimental setup with transparent monitor.

#### 2.2.2 Software

All testing was automated with a custom-written Python script running on a Raspberry Pi computer (*Raspberry Pi Model 4B, Raspberry Pi OS 2024-11-19-raspios-bookworm-arm64-full, available online:* https://raspberrypi.com/software/). The Raspberry Pi used precise hardware-timed signals via the pigpio library to control, through its GPIO pins, an electronic MOSFET switch (ALAMSCN, Shenzhen, China; Amazon ASIN B0924J4R47), which drove the LED panel. The Landolt C was drawn on the transparent LCD, and sufficient time (700 ms) was given for the liquid crystals to fully switch states before the LED backlight was triggered. The stimulus duration was thus strictly defined by the LED panel's on-time, independent of any limitations imposed by the LCD's temporal characteristics. The Freiburg Vision Test (FrACT; *frozen version: 1.5 2024-12-16, available online:* https://michaelbach.de/fract), was used to determine visual acuities efficiently using Landolt-C optotypes with eight possible orientations. A staircase procedure targeting 56.25% correct responses was used, with the optotype size controlled by the “Best PEST” algorithm (Lieberman and Pentland, 1982). The Python script handled the random selection of the conditions, triggered their execution in the FrACT, and recorded the resulting data. Reaction time from stimulus presentation to response entry was recorded for each optotype presentation but was not included in the present analysis.

### 2.3 Initial instructions

The method for determining visual acuity was explained to the participants, emphasizing that for the threshold to be measured accurately also sub-threshold stimuli would be shown. For such cases, they were instructed to rely on their intuition or guess. The participants received the same guidance for the different luminances—if a visual stimulus was too weak or not perceived at all, they should guess. They had as much time as they needed to enter their response. No correctness feedback was provided but participants were informed that optotype size was adjusted according to their preceding responses and response correctness.

### 2.4 Procedure

All conditions were measured twice in a randomized order in a single session. The session began with a practice run using a presentation duration of 50 ms at the maximum luminance. This familiarized participants with the sequence of the auditory cue and the subsequent flashed optotype, allowing them to blink during the cue and avoid blinking during stimulus presentation. Participants also practiced using the tactile markings on the numeric keypad without looking away from the monitor. Participants were allowed to take breaks ad libitum. A break of at least five minutes was taken after the first half of the session.

#### 2.4.1 Reference acuity

To determine a reference visual acuity with unconstrained stimulus duration, the FrACT was used with the recommended 18 optotype presentations (Bach, 2025). Two variants were tested: the standard variant, with black Landolt-C stimuli on a white background, and a variant with inverted contrast, consisting of white Landolt-C stimuli on a black background.

#### 2.4.2 Flashed-stimulus measurements

The optotypes were presented white-on-black to reduce changes in overall luminance when an optotype was presented and to minimize adaptation. As a result, only the white Landolt-C stimulus appeared on the previously black screen. When the LCD screen was not illuminated from behind by the LED panel for a defined duration, the optotype remained invisible. The illuminating flash was preceded by an audio signal 700 ms prior. For the flashed-stimulus measurements, the number of presentations was reduced from 18 to 14 to limit the session duration to approximately 90 min, a duration that had proved feasible in our previous comparable psychophysical experiments. Each run started with an acuity grade of 0.5 logMAR. The maximum size of a Landolt-C was limited to the boundaries of the visible part of the monitor.

We refrained from showing a mask stimulus following the optotype, as backward masking would negatively impact the recognizability of the stimulus (Raab, 1963). Instead, we accepted potentially occurring afterimages as a natural consequence of stimulation and a possibly mediating factor in stimulus recognition (Brindley, 1959). Between trials, the LED backlight was switched off, leaving the display completely black. The resulting stimulus-free interval was determined by the participant's response time. No additional fixed adaptation period was imposed to avoid prolonging the experiment and potentially increasing participant fatigue.

#### 2.4.3 Stimulus conditions

Visual acuities were measured for 25 fixed stimulus conditions comprising all combinations of five optotype luminances (3.3–53.4 cd/m^2^) and five presentation durations (0.125–512 ms). Each luminance–duration pair belonged to one of five flash strength levels spanning a 256-fold range (6.7, 26.7, 106, 427, and 1,708 cd·s/m^2^×10^−3^). Flash Strength Level 2 was generated by increasing the durations of Level 1 by a factor of four, while the luminances remained fixed. The stimulus luminance was controlled via grayscale values of the optotypes. Starting from the maximum luminance, each subsequent level was approximately halved. Verification with a luminance meter (LS100, Konica Minolta, Osaka, Japan) indicated luminances of 53.4, 26.8, 13.4, 6.7, and 3.3 cd/m^2^. Even the darkest gray relative to the black background had a Weber contrast (Bach et al., 2017) above 0.98. The covered range was intended to remain above the perceptual threshold, ensuring that the presence of the stimulus was detectable in all conditions to limit the task to discrimination rather than detection while avoiding saturation effects, i.e., high stimulus levels at which the acuity no longer differs between conditions. Pilot measurements indicated that these values represent a suitable compromise. Our experiment effectively provided a two-dimensional mapping of visual acuity across luminance levels and stimulus durations.

### 2.5 Analysis

All participants completed 54 runs (2 × 2 reference acuity runs + 2 × 25 flashed-stimulus measurements). All data (available under: https://doi.org/10.6084/m9.figshare.32790444) were automatically logged and later analyzed in Python (v3.11.2; Python Software Foundation, Wilmington, DE, USA) using the packages pandas, NumPy, Matplotlib, and Seaborn. The non-parametric Friedman test was used to assess differences between conditions across multiple related measurements. For pairwise comparisons, we used resampling methods (10,000 samples) to estimate statistical significance via permutation tests and confidence intervals via bootstrapping. All tests were conducted at a significance level of α = 0.05, with multiple comparisons controlled using the Bonferroni–Holm method (Holm, 1979).

## 3 Results

### 3.1 Test-retest reliability of the procedure

Figure 2 shows all results of the flashed-stimulus measurements in a test-retest scatter plot comparing the data of the first half with the second half. Closer alignment of the data points with the identity line indicates narrower limits of agreement. The acuity during the second run was slightly better (mean test-retest difference = −0.034 logMAR, SD = 0.153 logMAR). The limits of agreement ranged from −0.335 logMAR (lower) to +0.266 logMAR (upper).

**Figure 2:**
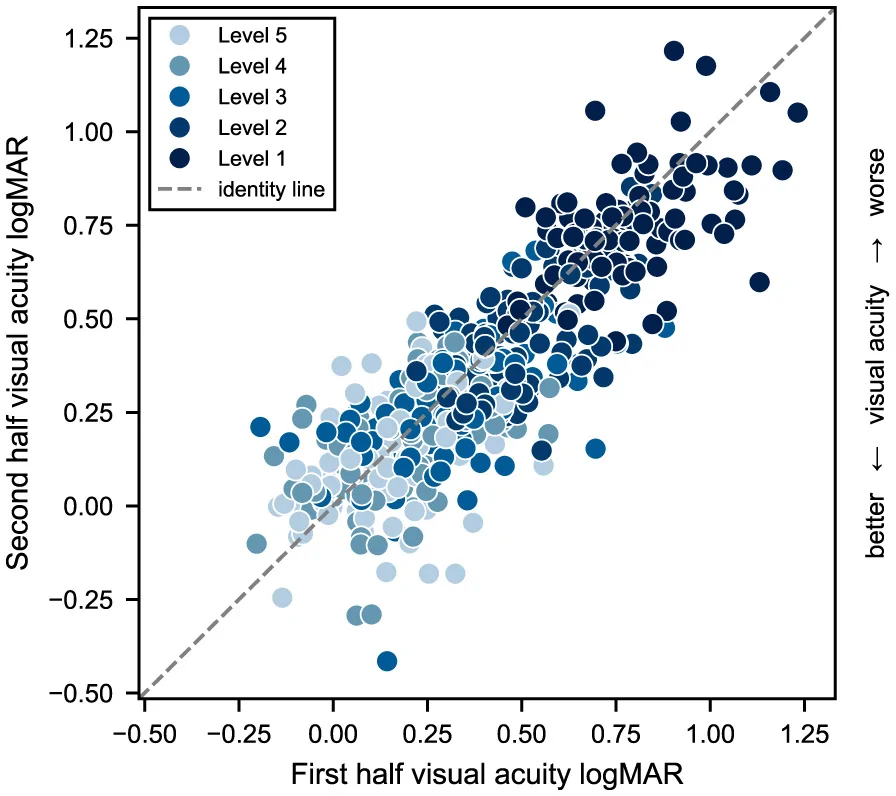
All flashed-stimulus measurements from all participants arranged in a test-retest scatter plot. Each visual acuity is compared between the first and second half. The data points are color-coded according to flash strength level. The dashed gray line represents the identity line.

### 3.2 Reference acuity for unlimited duration

The group mean for the reference acuity with normal contrast polarity was −0.083 logMAR (SD = 0.102 logMAR). For the inverted-contrast configuration as used for the flashed-stimulus measurements, the mean acuity was −0.080 logMAR (SD = 0.126 logMAR).

### 3.3 Overview of fixed conditions

The mean visual acuity of all participants for each of the 25 combinations of duration and luminance is shown in Figure 3. The combination with the worst visual acuity was *L* = 3.3 cd/m^2^ and *t* = 2 ms with +0.779 logMAR. The best visual acuity with +0.039 logMAR was at *L* = 3.3 cd/m^2^ and *t* = 512 ms. While acuity improved with increasing flash strength (smaller logMAR values), conditions with the same flash strength resulted in nearly identical acuity outcomes. In Figure 3, this manifests as a pattern of downward-diagonally similar colors. The largest pairwise difference in mean visual acuity within any one flash strength level (diagonal) was consistently below 0.1 logMAR (Level 1, 0.04 logMAR; Level 2, 0.08 logMAR; Level 3, 0.05 logMAR; Level 4, 0.07 logMAR). An exception is Level 5 (0.25 logMAR), where an improvement of acuity outcomes was noticeable for longer durations as also illustrated in Figure 4.

**Figure 3:**
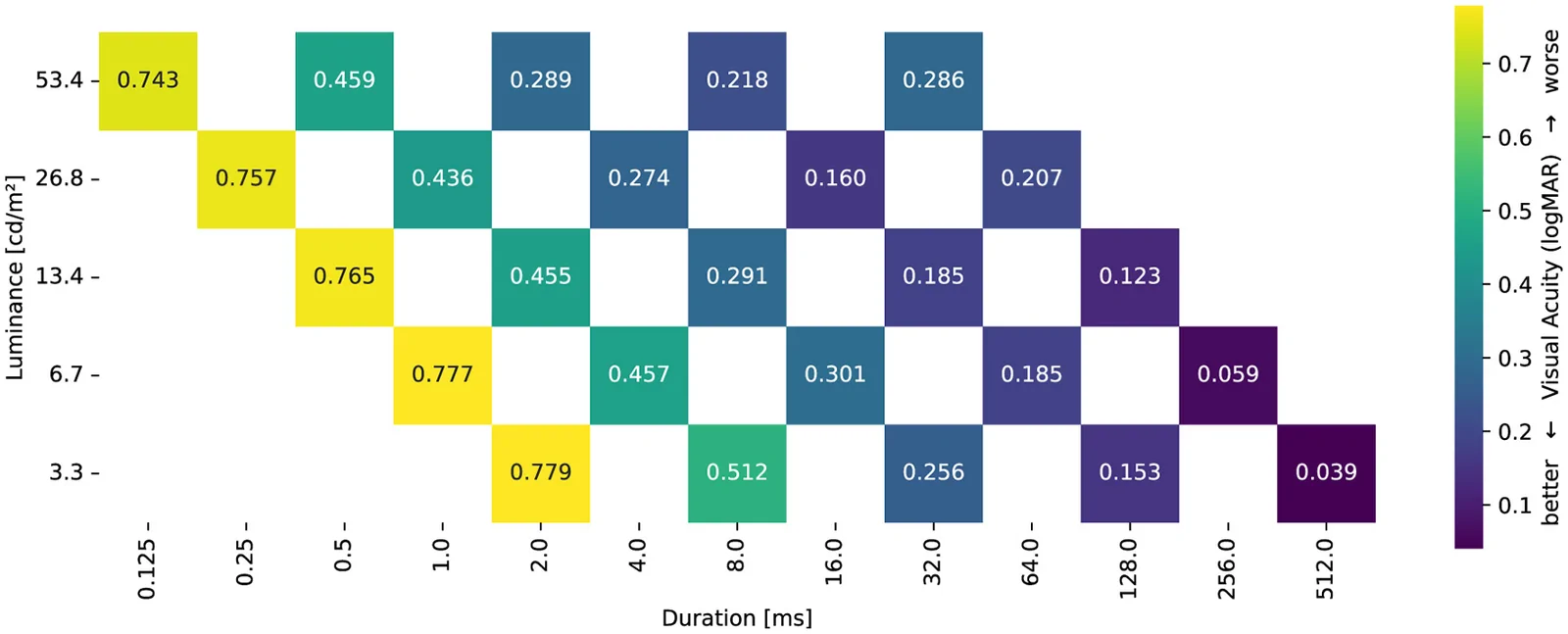
Overview of visual acuities (VA) for flashed-stimulus measurements. Each square represents the mean VA of all participants for a given pair of flash duration and luminance. The numerical value in each square indicates the visual acuity in logMAR visualized also by the color, where yellow corresponds to poorer visual acuity (higher logMAR) and dark purple corresponds to better visual acuity (lower logMAR). Conditions of equal flash strength (product of duration and luminance) align along diagonals running from top-left to bottom-right. The leftmost diagonal corresponds to the lowest flash strength and consistently shows the poorest acuity values, while the rightmost diagonal represents the highest flash strength and includes the best acuity performance.

**Figure 4:**
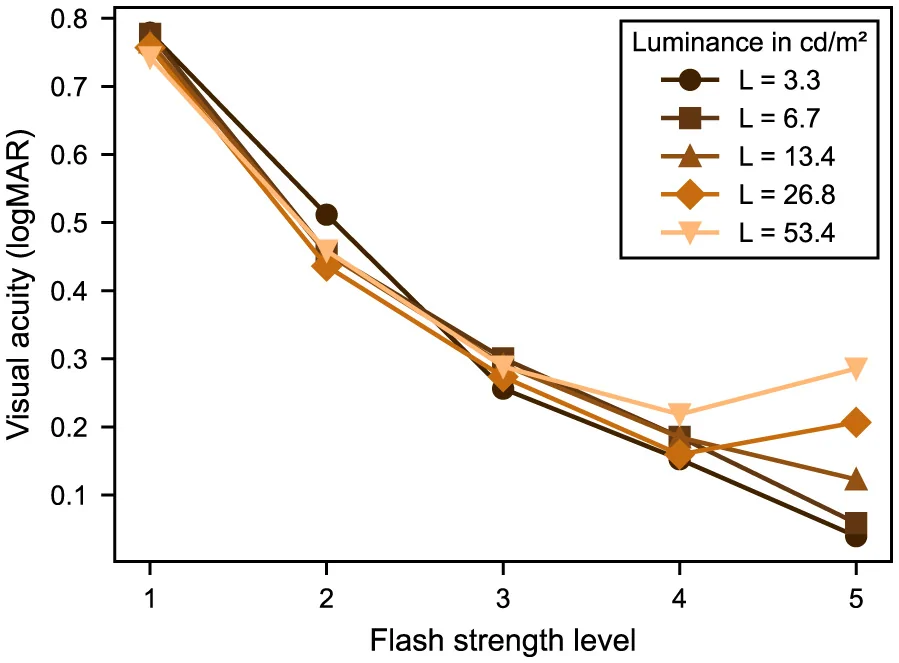
Visual acuity (logMAR) as a function of flash strength. The abscissa shows five flash strength levels corresponding to 6.7, 26.7, 106.8, 427.0, and 1,708.2 cd · s/m^2^ × 10^−3^. Each point represents the mean visual acuity across 19 participants for one luminance and flash strength. For the highest Flash Strength Level, acuities for the two highest luminances deviate from the general pattern.

### 3.4 Bloch-like time–luminance reciprocity

In order to assess whether visual acuity follows time–luminance reciprocity, we grouped the data by flash strength level. Within each group, we applied the non-parametric Friedman test for related measurements (Table 1). No significant differences were revealed for any of Levels 1–4 after Bonferroni–Holm correction, whereas Level 5 showed highly significant differences (Friedman statistic = 54.7, *p* < 0.001).

**Table 1:** Friedman test for differences within a level.

Level	Friedman statistic	*p*-Value
1	3.4	0.492
2	10.0	0.041
3	5.2	0.269
4	10.7	0.031
5	54.7	< 0.001^*^

^*^Significant after Bonferroni–Holm correction; reported *p* values are uncorrected.

To further investigate these differences within Level 5, we performed pairwise permutation tests for adjacent luminance steps with confidence intervals computed via bootstrapping (Table 2). Only the lowest step from 3.3 to 6.7 cd/m^2^ showed no significant change, while the comparisons 6.7 cd/m^2^ → 13.4 cd/m^2^, 13.4 cd/m^2^ → 26.8 cd/m^2^, and 26.8 cd/m^2^ → 53.4 cd/m^2^ all revealed significant logMAR increases (worse visual acuity).

**Table 2:** Differences within Level 5.

*L*_*a*_ [cd/m^2^]	*L*_*b*_ [cd/m^2^]	ΔVA(VA_*a*_−VA_*b*_) [CI]	*p*-Value
3.3	6.7	−0.019 [−0.063, 0.022]	0.399
6.7	13.4	−0.064 [−0.101, −0.026]	0.003^*^
13.4	26.8	−0.084 [−0.133, −0.034]	0.005^*^
26.8	53.4	−0.079 [−0.127, −0.032]	0.006^*^

Two-sided permutation test results for adjacent luminance levels *L*_*a*_ and *L*_*b*_ within Flash Strength Level 5. Shown are the mean differences in visual acuity, ΔVA, 95% confidence intervals, and uncorrected *p* values.

^*^Significant after Bonferroni–Holm correction within this flash strength, deliberately not accounting for the multiple Friedman tests that have been performed previously across flash strengths.

Providing a different perspective on the data, the aforementioned pattern of results is confirmed by Figure 5, showing that within each flash strength level visual acuities were very similar for Levels 1–4. At constant flash strength, stimulus duration and luminance covary inversely, such that changes along the abscissa reflect joint variations in both parameters rather than duration alone.

**Figure 5:**
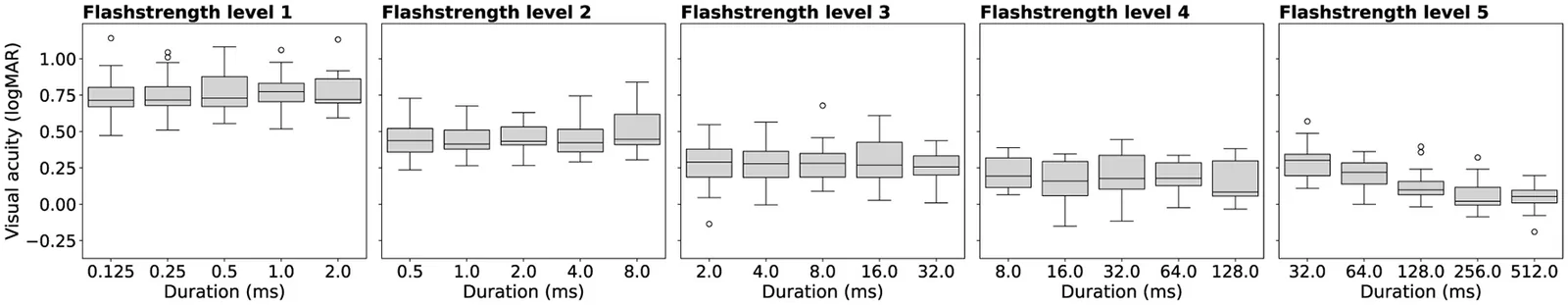
Visual acuity (logMAR) as a function of flash duration for Flash Strength Levels 1–5. Note the differences in abscissa scaling. The ordinate shows logMAR, with smaller values indicating better acuity. Schematic boxplots are used to represent interquartile ranges and medians (Dawson, 2011). While there are only small differences between acuity medians for the lower four flash strength levels, values for the highest flash strength level improve with longer durations.

## 4 Discussion

The main findings of the present study are as follows:

1. Our setup provides robust visual acuity measurements across various luminance–duration combinations, extending into the sub-millisecond range.
2. As anticipated, mean visual acuity consistently improved with increasing flash strength.
3. Visual acuity increased irrespective of whether flash strength was increased by lengthening the display time or increasing the luminance within Flash Strength Levels 1–4 across 0.125–128 ms.

Below the critical duration, light perception depends only on the product of luminance and duration (Bloch's Law) (Bloch, 1885). This reciprocity in human vision resembles the earlier Bunsen-Roscoe Law of photochemistry, which described the response of analog photographic emulsions. (Bunsen and Roscoe, 1863). We found that within each of the Flash Strength Levels 1–4, with durations up to 128 ms, visual acuity remained stable (deviations below 0.08 logMAR) for changes in duration and luminance when the flash strength was kept constant. This is consistent with time–luminance reciprocity and parallels Bloch's Law.

Bloch-like reciprocity across several octaves of duration implies a robust reciprocity between stimulus luminance and duration. However, the slightly longer integration times observed in shape recognition compared to simple detection may reflect the cumulative dynamics of hierarchical processing. While retinal integration occurs within tens of milliseconds, cortical spatiotemporal pooling of these signals to form a coherent percept of a shape likely extends the effective temporal window. This process allows the visual system to integrate features across both space and time.

Regarding the applicability of a Bloch-like law to visual acuity, our findings are in qualitative agreement with those of Niwa and Tokoro (1997). In their study, acuity was measured using a modified tachistoscope presenting black Landolt Cs on a white background while systematically varying exposure duration and background luminance. They found that for visual acuity worse than 0.16 logMAR, acuity can be described as a function of the product of stimulus duration and luminance. However, for better acuity, the applicability was questioned due to incomplete or absent temporal summation or measurement limitations, as small changes in the Landolt-C gap size resulted in large variability in the data. In our data the conditions with the best acuity results were within the strongest flash strength level. At Level 5 (32–512 ms), where the longest durations likely exceed the critical duration (*t*_c_) for complete temporal summation, visual acuity improved substantially with increasing duration, ranging from 0.29 to 0.04 logMAR. At first, this seems counterintuitive: if temporal summation ceases beyond *t*_c_, extended duration at proportionally reduced luminance should decrease the perceived brightness and therefore worsen acuity. The fact that acuity instead improved statistically significantly between adjacent durations from 32 to 256 ms (32–64, 64–128, and 128–256 ms) indicates that in this range visual acuity cannot be explained solely by flash strength.

Several explanations are possible: with more time to inspect the optotype, observers may benefit from fluctuations in the tear film (Montés-Micó, 2007), accommodation (Charman and Heron, 1988), and cognitive processing (Heinrich et al., 2010). It is important to note again that within Level 5, longer durations are compensated by lower luminances. Therefore, any duration benefits must outweigh the effects of reduced luminance. Alternatively, the improvement could also reflect a decline in the opposite direction where the highest luminance conditions in Level 5 were too bright, making gap detection in the Landolt C more difficult. This interpretation is supported by the fact that the reduction in visual acuity between 8 and 32 ms at 53.4 cd/m^2^ and between 16 and 64 ms at 26.8 cd/m^2^ are the only conditions where increasing the duration at constant luminance led to worse visual acuity. Interestingly, participants did not report a subjective sensation of glare but instead characterized certain conditions as “washed out.” This suggests that the observed decline in visual acuity may reflect an increase in veiling luminance, leading to a functional degradation of the retinal image. For future investigations, Flash Strength Level 5 could be extended to lower luminance. This would also enable a more detailed investigation of probability summation over time by comparing deviations with the predictions from Gorea's double-integration model (Gorea and Tyler, 1986).

To keep the total session duration below 2 h, the number of trials during the flashed-stimulus measurements was reduced to 14, accepting slightly increased test-retest limits of agreement (LoA) as a trade-off. The LoAs in our experiment ranged from −0.335 logMAR (lower) to +0.266 logMAR (upper), which is larger than the 0.17 logMAR found by Bach (2025) with the recommended 18 trials. Despite this increase, the results still indicate that the procedure provides reliable estimates of visual acuity.

In contrast to presentation techniques used in most previous studies on the perception of very briefly displayed stimuli, our setup is easy to replicate by removing the backlight from a standard LCD monitor and replacing it with a computer-controlled LED panel. The methods established for the present study thus provide an avenue for investigating the interactions between retinal physiology and higher-level dynamic temporal factors in visual tasks that are more complex than simple detection of light.

In summary, we demonstrated across four octaves of luminance and three orders of magnitude in exposure duration that shape recognition, as operationalized via optotype visual acuity, is governed by temporal dynamics similar to those described by Bloch's Law for flash detection.

## Data Availability

The datasets presented in this study can be found in online repositories. The names of the repository/repositories and accession number(s) can be found below: https://doi.org/10.6084/m9.figshare.32790444.

## References

[B1] Amerio P. Cleeremans A. (2025). Bloch strikes back: a reassessment of the intensity-duration reciprocity. PsyArXiv. [Preprint]. doi: 10.31234/osf.io/b5yuv_v1

[B2] Bach M. (2025). Freiburg vision test (FrACT): optimal number of trials? Graefes Arch. Clin. Exp. Ophthalmol. 263, 437–442. doi: 10.1007/s00417-024-06638-z 39294391 PMC11868300

[B3] Bach M. Hoffmann M. B. Jägle H. Heinrich S. P. Schiefer U. Wesemann W. (2017). Kontrastsehen - Definitionen, Umrechnungen und Äquivalenztabelle. Der Ophthalmologe 114, 341–347. doi: 10.1007/s00347-016-0379-5 27785557

[B4] Barlow H. B. (1958). Temporal and spatial summation in human vision at different background intensities. J. Physiol. 141, 337–350. doi: 10.1113/jphysiol.1958.sp005978 13539843 PMC1358805

[B5] Bloch A.-M. (1885). Experiences sur la vision. C R Soc Biol. 37, 493–495.

[B6] Brindley G. S. (1959). The discrimination of after-images. J. Physiol. 147, 194–203. doi: 10.1113/jphysiol.1959.sp006234 13673397 PMC1357015

[B7] Bunsen R. Roscoe H. (1863). Photochemische Untersuchungen. Annalen der Physik 193, 529–562. doi: 10.1002/andp.18631931202

[B8] Charman W. Heron G. (1988). Fluctuations in accommodation: a review. Ophthalmic Physiol. Opt. 8, 153–164. doi: 10.1111/j.1475-1313.1988.tb01031.x 3062549

[B9] Dawson R. (2011). How significant is a boxplot outlier? J. Stat. Educ. 19:2. doi: 10.1080/10691898.2011.11889610

[B10] Gopiswaminathan A. V. Haldina J. Al-Nosairy K. O. Duval C. Z. Stolle F. H. Hoffmann M. B. . (2024). Objective visual acuity estimates in amblyopia are more accurate with optotype-based P300 than with VEP measurements. Transl. Vis. Sci. Technol. 13:30. doi: 10.1167/tvst.13.12.30 39688849 PMC11654770

[B11] Gorea A. (2015). A refresher of the original Bloch's Law Paper (Bloch, July 1885). i Perception 6:2041669515593043. doi: 10.1177/2041669515593043 27433317 PMC4934648

[B12] Gorea A. Tyler C. W. (1986). New look at Bloch's law for contrast. J. Opt. Soc. Am. A 3, 52–61. doi: 10.1364/JOSAA.3.000052 3950792

[B13] Greene E. (2013). Violation of Bloch's Law that specifies reciprocity of intensity and duration with brief light flashes. i-Perception 4:543. doi: 10.1068/i0619rep 25165512 PMC4129388

[B14] Greene E. Ogden R. T. (2013). Shapes displayed with durations in the microsecond range do not obey Bloch's law of temporal summation. i-Perception 4, 429–436. doi: 10.1068/i0602 24349700 PMC3859558

[B15] Heinrich S. P. Bach M. (2013). Resolution acuity versus recognition acuity with Landolt-style optotypes. Graefes Arch. Clin. Exp. Ophthalmol. 251, 2235–2241. doi: 10.1007/s00417-013-2404-6 23828648

[B16] Heinrich S. P. Krüger K. Bach M. (2010). The effect of optotype presentation duration on acuity estimates revisited. Graefes Arch. Clin. Exp. Ophthalmol. 248, 389–394. doi: 10.1007/s00417-009-1268-2 20076965

[B17] Holm S. (1979). A simple sequentially rejective multiple test procedure. Scand. J. Statist. 6, 65–70.

[B18] Kahneman D. (1964). Temporal summation in an acuity task at different energy levels-a study of the determinants of summation. Vis. Res. 4, 557–566. doi: 10.1016/0042-6989(64)90041-0 5888624

[B19] Lieberman H. R. Pentland A. P. (1982). Microcomputer-based estimation of psychophysical thresholds: the Best PEST. Behav. Res. Methods Instrum. 14, 21–25. doi: 10.3758/BF03202110

[B20] Loffler G. (2008). Perception of contours and shapes: low and intermediate stage mechanisms. Vis. Res. 48, 2106–2127. doi: 10.1016/j.visres.2008.03.006 18502467

[B21] Meister M. Berry M. J. (1999). The neural code of the retina. Neuron 22, 435–450. doi: 10.1016/S0896-6273(00)80700-X 10197525

[B22] Montés-Micó R. (2007). Role of the tear film in the optical quality of the human eye. J. Cataract Refract. Surg. 33, 1631–1635. doi: 10.1016/j.jcrs.2007.06.019 17720082

[B23] Mulholland P. J. Redmond T. Garway-Heath D. F. Zlatkova M. B. Anderson R. S. (2015). Estimating the critical duration for temporal summation of standard achromatic perimetric stimuli. Invest. Ophthalmol. Vis. Sci. 56, 431–437. doi: 10.1167/iovs.14-15304 25515580

[B24] Niwa K. Tokoro T. (1997). Measurement of temporal summation of visual acuity with use of modified tachistoscope. Jpn. J. Ophthalmol. 41, 403–408. doi: 10.1016/S0021-5155(97)00082-8 9509308

[B25] Raab D. H. (1963). Backward masking. Psychol. Bullet. 60, 118–129. doi: 10.1037/h0040543 13972804

[B26] Robson A. G. Frishman L. J. Grigg J. Hamilton R. Jeffrey B. G. Kondo M. . (2022). ISCEV Standard for full-field clinical electroretinography (2022 update). Doc. Ophthalmol. 144, 165–177. doi: 10.1007/s10633-022-09872-0 35511377 PMC9192408

[B27] Wang X.-J. (2012). Neural dynamics and circuit mechanisms of decision-making. Curr. Opin. Neurobiol. 22, 1039–1046. doi: 10.1016/j.conb.2012.08.006 23026743 PMC4065788

[B28] Yau K. W. (1994). Phototransduction mechanism in retinal rods and cones. The Friedenwald Lecture. Invest. Ophthalmol. Vis. Sci. 35, 9–32. 7507907

